# Supplementary material for: Implementation of MALDI-TOF Mass Spectrometry and Peak Analysis: Application to the Discrimination of Cryptococcus neoformans Species Complex and Their Interspecies Hybrids
Source: J Fungi (Basel). 2020 Dec 2;6(4):330. doi: 10.3390/jof6040330 (PMC7711916; doi:10.3390/jof6040330)
Supplement: Supplementary file 1 [file jof-06-00330-s001.zip › Supplementary_Figures_09.11.2020.pdf]

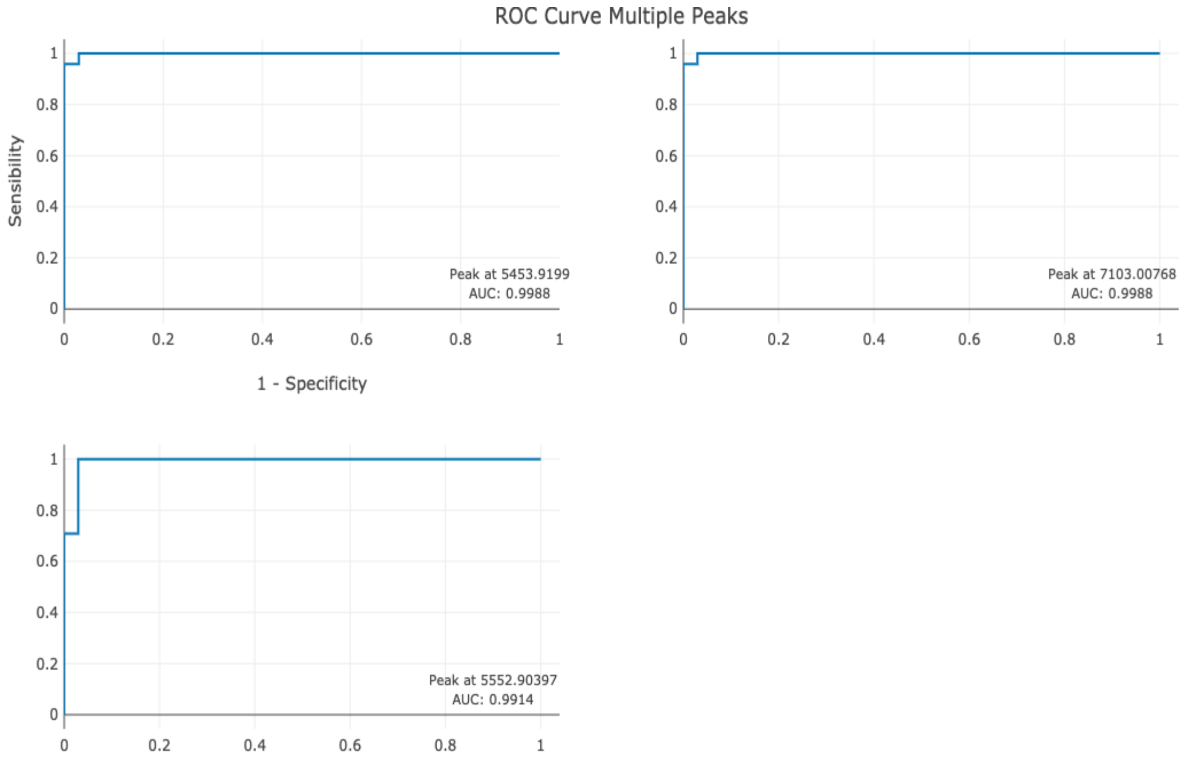

Figure S1. ROC Curve of MS peaks of *Cryptococcus spp.* according to the criteria for biomarkers search.

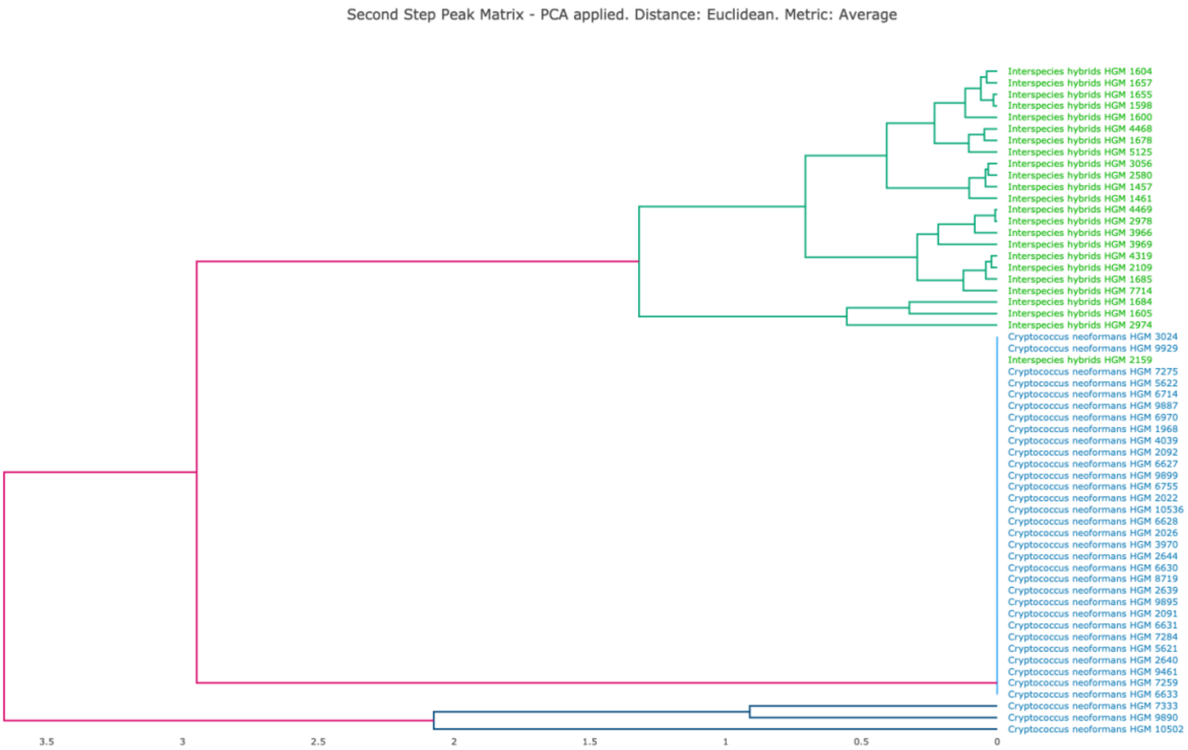

Figure S2. Discrimination between *C. neoformans* and the interspecies hybrids using the two-step classification method.

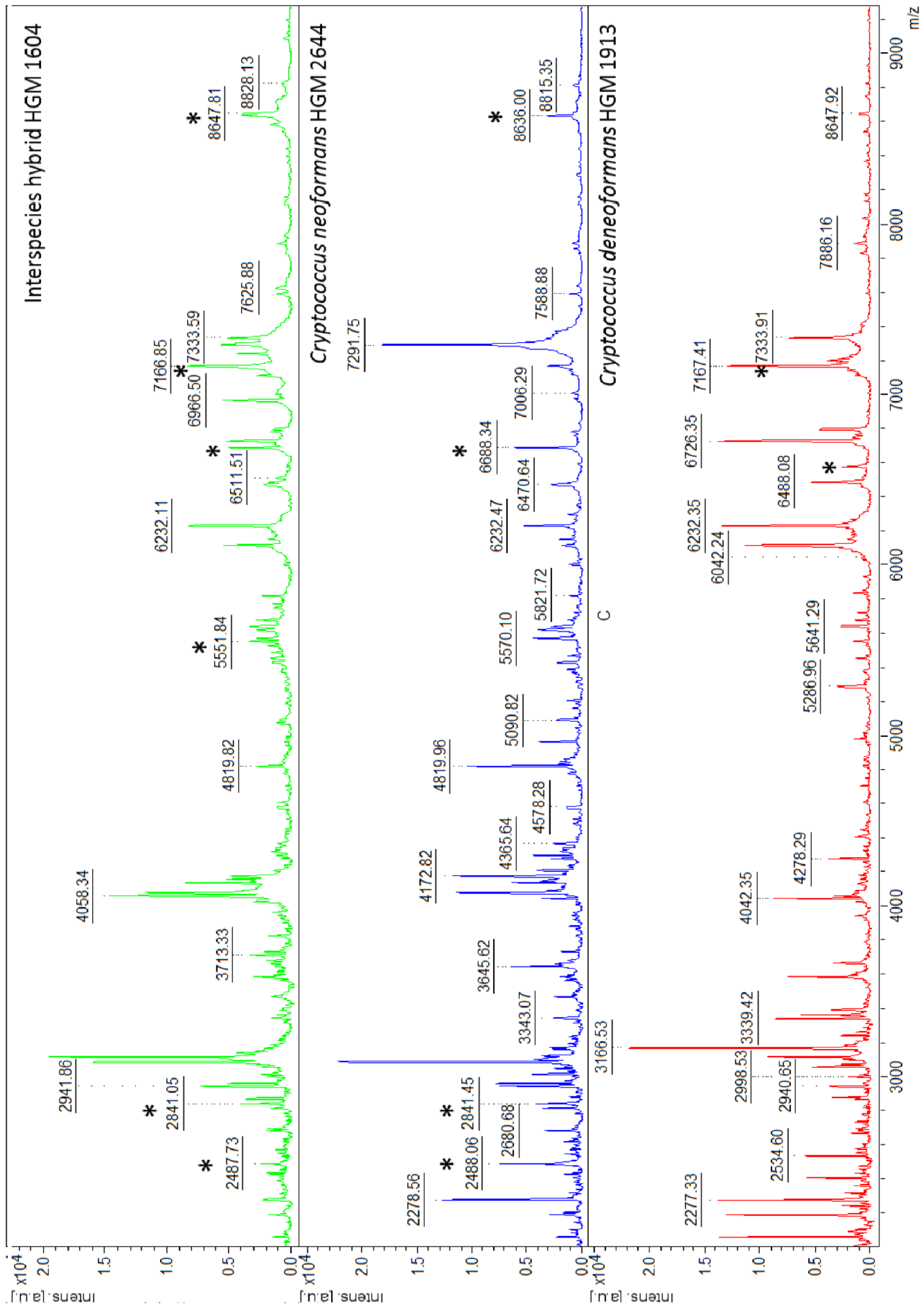

Figure S3. Representative protein spectra from the three Cryptococcus species analyzed in this study. Species-specific peaks with intensity above 500 arbitrary units (a. u.) are marked with asterisks (*). The complete list of species-specific peaks can be found in Table 2.
