# Supplementary material for: Implementation of MALDI-TOF Mass Spectrometry and Peak Analysis: Application to the Discrimination of Cryptococcus neoformans Species Complex and Their Interspecies Hybrids
Source: J Fungi (Basel). 2020 Dec 2;6(4):330. doi: 10.3390/jof6040330 (PMC7711916; doi:10.3390/jof6040330)
Supplement: Supplementary file 1 [file jof-06-00330-s001.zip › Supplementary_Tables_rev.pdf]

Patient Number	Strain Name	Clinical Sample of isolation	Year	AFLP Genotype	Identification by AFLP	Identification by ITS1-5.8S-ITS2 sequencing
1	HGM 10502	Cerebrospinal fluid	2007	1	*C. neoformans*	*C. neoformans*
2	HGM 10536	Cerebrospinal fluid	2007	1	*C. neoformans*	*C. neoformans*
3	HGM 2022	Cerebrospinal fluid	1995	1	*C. neoformans*	*C. neoformans*
4	HGM 2026	Blood	1995	1	*C. neoformans*	*C. neoformans*
**5**	HGM 2091	Lung biopsy	1995	1	*C. neoformans*	*C. neoformans*
**5**	HGM 2092	Cerebrospinal fluid	1995	1	*C. neoformans*	*C. neoformans*
6	HGM 2639	Blood	1997	1	*C. neoformans*	*C. neoformans*
7	HGM 2640	Blood	1997	1	*C. neoformans*	*C. neoformans*
8	HGM 2644	Urine	1997	1	*C. neoformans*	*C. neoformans*
9	HGM 3024	Cerebrospinal fluid	1998	1	*C. neoformans*	*C. neoformans*
10	HGM 4039	Cerebrospinal fluid	1999	1	*C. neoformans*	*C. neoformans*
11	HGM 6627	Cerebrospinal fluid	2003	1	*C. neoformans*	*C. neoformans*
12	HGM 6714	Lung biopsy	2003	1	*C. neoformans*	*C. neoformans*
13	HGM 6755	Blood	2003	1	*C. neoformans*	*C. neoformans*
14	HGM 7259	Cerebrospinal fluid	2004	1	*C. neoformans*	*C. neoformans*
15	HGM 7275	Cerebrospinal fluid	2004	1	*C. neoformans*	*C. neoformans*
16	HGM 7284	Cerebrospinal fluid	2004	1	*C. neoformans*	*C. neoformans*
17	HGM 7333	Cerebrospinal fluid	2004	1	*C. neoformans*	*C. neoformans*
18	HGM 8719	Cerebrospinal fluid	2005	1	*C. neoformans*	*C. neoformans*
19	HGM 10916	Blood	2007	2	*C. deneoformans*	*C. deneoformans*
20	HGM 1459	Cerebrospinal fluid	1994	2	*C. deneoformans*	*C. deneoformans*
21	HGM 1888	Cerebrospinal fluid	1995	2	*C. deneoformans*	*C. deneoformans*
22	HGM 1913	Bronchoalvelolar lavage	1995	2	*C. deneoformans*	*C. deneoformans*
23	HGM 1972	Blood	1995	2	*C. deneoformans*	*C. deneoformans*
24	HGM 2143	Cerebrospinal fluid	1996	2	*C. deneoformans*	*C. deneoformans*
25	HGM 4760	Cerebrospinal fluid	2001	2	*C. deneoformans*	*C. deneoformans*
26	HGM 1457	Cerebrospinal fluid	1994	3	Interspecies hybrid	*C. deneoformans*
27	HGM 1461	Blood	1994	3	Interspecies hybrid	*C. deneoformans*
28	HGM 1598	Blood	1994	3	Interspecies hybrid	*C. deneoformans*
29	HGM 1600	Bone marrow	1994	3	Interspecies hybrid	*C. deneoformans*
**30**	HGM 1604	Blood	1994	3	Interspecies hybrid	*C. deneoformans*
**30**	HGM 1605	Cerebrospinal fluid	1994	3	Interspecies hybrid	*C. deneoformans*

31	HGM 1655	Cerebrospinal fluid	1995	3	Interspecies hybrid	*C. deneoformans*
32	HGM 1657	Cerebrospinal fluid	1995	3	Interspecies hybrid	*C. deneoformans*
33	HGM 1678	Cerebrospinal fluid	1995	3	Interspecies hybrid	*C. deneoformans*
34	HGM 1684	Cerebrospinal fluid	1995	3	Interspecies hybrid	*C. deneoformans*
35	HGM 1685	Blood	1995	3	Interspecies hybrid	*C. deneoformans*
36	HGM 1767	Cerebrospinal fluid	1995	3	Interspecies hybrid	*C. deneoformans*
37	HGM 1768	Blood	1995	3	Interspecies hybrid	*C. deneoformans*
38	HGM 1784	Cerebrospinal fluid	1995	3	Interspecies hybrid	*C. deneoformans*
39	HGM 2109	Cerebrospinal fluid	1995	3	Interspecies hybrid	*C. deneoformans*
40	HGM 2159	Blood	1996	3	Interspecies hybrid	*C. deneoformans*
41	HGM 2456	Blood	1996	3	Interspecies hybrid	*C. deneoformans*
42	HGM 2580	Cerebrospinal fluid	1997	3	Interspecies hybrid	*C. deneoformans*
43	HGM 2974	Cerebrospinal fluid	1998	3	Interspecies hybrid	*C. deneoformans*
44	HGM 2978	Blood	1998	3	Interspecies hybrid	*C. deneoformans*
45	HGM 3056	Cerebrospinal fluid	1998	3	Interspecies hybrid	*C. deneoformans*
46	HGM 3966	Blood	1999	3	Interspecies hybrid	*C. deneoformans*
47	HGM 3969	Blood	1999	3	Interspecies hybrid	*C. deneoformans*
48	HGM 4319	Blood	2000	3	Interspecies hybrid	*C. deneoformans*
**49**	HGM 4468	Blood	2000	3	Interspecies hybrid	*C. deneoformans*
**49**	HGM 4469	Cerebrospinal fluid	2000	3	Interspecies hybrid	*C. deneoformans*
50	HGM 5125	Blood	2001	3	Interspecies hybrid	*C. deneoformans*
51	HGM 5621	Cerebrospinal fluid	2002	3	Interspecies hybrid	*C. deneoformans*
52	HGM 7714	Cerebrospinal fluid	2004	3	Interspecies hybrid	*C. deneoformans*
53	HGM 1968	Blood	1995	1B	*C. neoformans*	*C. neoformans*
54	HGM 3970	Blood	1999	1B	*C. neoformans*	*C. neoformans*
55	HGM 5622	Cerebrospinal fluid	2002	1B	*C. neoformans*	*C. neoformans*
56	HGM 6628	Wound	2003	1B	*C. neoformans*	*C. neoformans*
57	HGM 6630	Blood	2003	1B	*C. neoformans*	*C. neoformans*
58	HGM 6631	Wound	2003	1B	*C. neoformans*	*C. neoformans*
59	HGM 6633	Cerebrospinal fluid	2003	1B	*C. neoformans*	*C. neoformans*
60	HGM 6970	Cerebrospinal fluid	2003	1B	*C. neoformans*	*C. neoformans*

61	HGM 9461	Cerebrospinal fluid	2006	1B	*C. neoformans*	*C. neoformans*
62	HGM 9887	Bronchoalvelolar lavage	2007	1B	*C. neoformans*	*C. neoformans*
63	HGM 9890	Bronchoaspirate	2007	1B	*C. neoformans*	*C. neoformans*
64	HGM 9895	Sputum	2007	1B	*C. neoformans*	*C. neoformans*
65	HGM 9899	Bronchoaspirate	2007	1B	*C. neoformans*	*C. neoformans*
66	HGM 9901	Blood	2007	1B	*C. neoformans*	*C. neoformans*
67	HGM 9929	Sputum	2007	1B	*C. neoformans*	*C. neoformans*

Table S1. Detailed information about the *Cryptococcus* strains included in this study.

Baseline Subtraction	Tophat filter<br>Factor: 0.02
Noise Reduction	Saviztky-Golay filter<br>-Window length: 11<br>-Polynomial order: 3
Alignment	Allowed shift: Medium<br>Mass tolerance for aligning the spectra:<br>-Linear Mass tolerance: 2000 ppm
Peaks Finding	By threshold<br>-Peak threshold: 0.01<br>-Constant Mass tolerance: 0.5Da<br>-Linear Mass tolerance: 2000ppm<br>-Analysis Data: Peak intensity

Table S2. Pre-treatment of raw data spectra for the biomarkers search.

Baseline Subtraction	Tophat filter<br>Factor: 0.02
Noise Reduction	Saviztky-Golay filter<br>-Window length: 11<br>-Polynomial order: 3
Alignment	Allowed shift: Medium<br>Mass tolerance for aligning the spectra:<br>-Linear Mass tolerance: 2000 ppm
Peaks Finding	By mass position<br>-Constant Mass tolerance: 0.5Da<br>-Linear Mass tolerance: 2000ppm
Merge	Mass tolerance for aligning the peaks<br>- Constant Mass tolerance: 1Da<br>- Linear Mass tolerance: 2000ppm
Normalization	-Total Ion Count (TIC)

Table S3. Parameters applied for the construction of the peak matrix.

A

PC 0 (46.73%)

Score: 100 %

Actual / Predicted	Deneoformans	Hybrid/Neoformans	% Correct
Deneoformans	7	0	100 %
Hybrid/Neoformans	0	58	100 %
	100 %	100 %	100 %

Confusion Matrix

k-fold Cross Validation

10-fold cross validation

Actual / Predicted	Deneoformans	Hybrid/Neoformans	% Correct
Deneoformans	7	0	100 %
Hybrid/Neoformans	0	58	100 %
	100 %	100 %	100 %

Confusion Matrix

B

Score: 98.46 %

From / to	Deneoformans	Interspecies hybrids	Neoformans	% Correct
Deneoformans	7	0	0	100 %
Interspecies hybrids	0	23	1	95.83 %
Neoformans	0	0	34	100 %
				98.46 %

Confusion Matrix

k-fold Cross Validation

10-fold cross validation

From / to	Deneoformans	Interspecies hybrids	Neoformans	% Correct
Deneoformans	6	1	0	85.71 %
Interspecies hybrids	0	23	1	95.83 %
Neoformans	0	0	34	100 %
				96.92 %

Confusion Matrix

Table S4. Score and validation of the SVM analysis applied to (A) the two-step model with 5 biomarkers and (B) the one-step model with 5 biomarkers. K=10
